# Autosomal Dominant Vitreoretinochoroidopathy With a Novel BEST1 Mutation and a Review of Reported Mutations

**DOI:** 10.7759/cureus.32990

**Published:** 2022-12-27

**Authors:** Jack Komro, Sarah Skender, Bing X Ross, Xihui Lin

**Affiliations:** 1 Ophthalmology, Ascension Macomb-Oakland Hospital/Ascension Eye Institute, Warren, USA; 2 Ophthalmology, Kresge Eye Institute/Wayne State University School of Medicine, Detroit, USA

**Keywords:** case report, review, mutation, best1, autosomal dominant vitreoretinochoroidopathy

## Abstract

Here we describe a patient with atypical presentation of autosomal dominant vitreoretinochoroidopathy (ADVIRC) with a novel missense mutation in BEST1 gene and briefly review reported ADVIRC-associated genetic mutations. The patient is a 71-year-old African American female who presented with progressively worsening blurry vision bilaterally over the course of 40 years, with significant deterioration in both peripheral and central vision in the past five years. Her anterior segment exam was unremarkable. Fundoscopic examination showed confluent, demarcated areas of pigmentary chorioretinal atrophy in the mid-periphery of the retina with sparing of the macula in both eyes. Optical coherence tomography (OCT) of the lesions revealed flattening of the fovea with an elevation of the inner retinal structures and outer plexiform layer, and peripheral retinal thinning and loss of retinal structures with choroid hyperreflectivity, consistent with peripheral chorioretinal atrophy. Genetic testing identified a heterozygous c.830C>T, p.(T277M) mutation located on exon 7 of the BEST1 gene. This patient represents an atypical presentation of ADVIRC with more posterior involvement, and this case is associated with a novel missense mutation in the BEST1 gene.

## Introduction

Autosomal dominant vitreoretinochoroidopathy (ADVIRC) is a rare disorder with a prevalence of 1:1,000,000 [1]. Patients affected with ADVIRC usually experience night vision disturbances and visual field abnormalities during adulthood [1]. Visual acuity thereafter would slowly deteriorate with a gradually constricted visual field leading to severe visual impairment in the advanced stage of the disease. Fundus exam usually shows a sharply demarcated circumferential hyperpigmented band in the peripheral retina with a normal central retina at the time of diagnosis [1]. Chorioretinal atrophy and progressive involvement of the central retina are seen in the advanced stage of the disease. A hallmark feature is the abrupt transition from the normal retina to the atrophic retina in the mid-periphery [1]. Other ocular features include fibrillar vitreous condensation, retinal neovascularization, and varied anterior segment abnormalities, such as microcornea, microphthalmia, angle-closure glaucoma, and presenile cataract [1].

ADVIRC follows an autosomal dominant inheritance pattern of bestrophin 1 (BEST1) gene mutations [2]. The BEST1 gene is located on chromosome 11q13, and its RNA transcript has 11 exons. The encoded BEST1 protein is a pentameric calcium-activated anion channel primarily located in the basolateral side of retinal pigment epithelium (RPE) [3-5]. The missense mutations in BEST1 gene in association with ADVIRC are believed to affect the functions of the BEST1 protein channel in RPE, leading to chorioretinal atrophy [5]. A few diseases other than ADVIRC resulting from BEST1 gene mutations include Best Disease, Adult Vitelliform Macular Dystrophy, and Retinitis Pigmentosa [1]. 

Due to the low prevalence and high interfamilial and intrafamilial phenotypic variability, the diagnosis of ADVIRC is usually difficult and requires genetic testing. Here we reported an atypical presentation of ADVIRC with a novel missense mutation in BEST1 gene, which is likely pathogenic.

## Case presentation

A 71-year-old African American female presented to our retina clinic with progressively worsening blurry vision bilaterally over the course of 40 years. The patient complained of a significant decline in her night, peripheral, and central vision in the past five years, with central visual acuity (VA) deteriorating from 20/60 in both eyes to 20/100 in the right eye and 20/80 in the left eye. She denied a family history of retinitis pigmentosa. Her past medical history is pertinent for type 2 diabetes mellitus without signs of diabetic retinopathy, hypertension with hypertensive retinopathy, one episode of uveitis at the age of 58, and cataract extraction with intraocular lens placement in both eyes at the age of 59.

**Figure 1:**
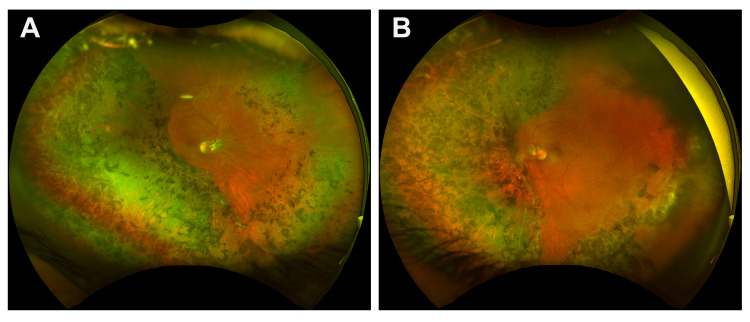
Color Fundus Photographs of the Reported Patient Right (A) and left (B) eyes show confluent, demarcated areas of pigmentary chorioretinal atrophy in the near periphery of the retina with sparing of the macula.

**Figure 2:**
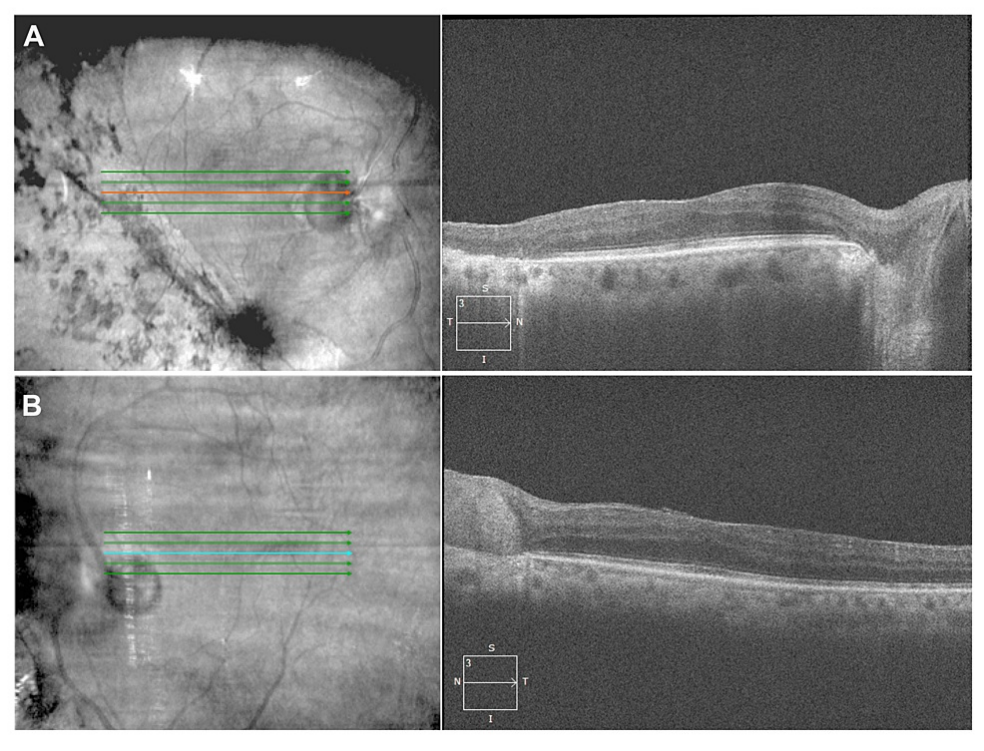
Optical Coherence Tomography of the Reported Patient (A) Right eye. Flattening of the fovea with an elevation of the inner retinal structures and outer plexiform layer. Near peripheral retinal thinning and loss of retinal structures with choroid hyperreflectivity, consistent with chorioretinal atrophy. (B) Left eye. Epiretinal membrane, mild flattening of the fovea with an elevation of the inner retinal structures and outer plexiform layer, and disruption of the outer plexiform layer in the parafoveal region.

The patient had constricted visual fields in both eyes on confrontational visual field testing. Her anterior segment exam was unremarkable except the right pupil was irregular with a sluggish response to light. The fundoscopic examination was significant for confluent, demarcated areas of chorioretinal scarring with intraretinal pigment migration band in the peripheral retina more apparent temporally in the right eye and nasally in the left eye (Figure 1). The macula regions were relatively spared in both eyes (Figure 1). Optical coherence tomography (OCT) in the right eye revealed flattening of the fovea with an elevation of the inner retinal structures and outer plexiform layer, and peripheral retinal thinning and loss of retinal structures with choroid hyperreflectivity, consistent with peripheral chorioretinal atrophy (Figure 2A). The left eye showed an epiretinal membrane, mild flattening of the fovea with an elevation of the inner retinal structures and outer plexiform layer, and disruption of the outer plexiform layer in the parafoveal region (Figure 2B). The prominent area of involvement was located temporally in the right eye and nasally in the left eye.

Given the overall clinical picture, we suspect this is an atypical presentation of ADVIRC. Therefore, genetic testing with the Invitae Inherited Retinal Disorders Panel (evaluation of 248 genes) was performed. A heterozygous c.830C>T, p.(T277M) mutation located on exon 7 of the BEST1 gene was identified. We believe this mutation represents a new pathogenic locus in the BEST1 gene associated with ADVIRC.

## Discussion

Genetic mutations to the BEST1 gene result in a spectrum of five disorders collectively named bestrophinopathies: Best Disease, Adult Vitelliform Macular Degeneration (AVMD), Autosomal Recessive Bestrophinopathy (ARB), Retinitis Pigmentosa (RP), and Autosomal Dominant Vitreoretinochoroidopathy (ADVIRC) [1,6]. ADVIRC is a rare disorder with varied phenotypic presentations, leading to difficulty in diagnosis. Here we presented a case of an atypical presentation of ADVIRC with a novel BEST1 gene mutation that has not been associated with ADVIRC previously.

The characteristic feature of ADVIRC is a hyperpigmented band in the peripheral retina, with central retina sparing in the early stage of the disease. As the disease progresses, chorioretinal atrophy develops and progresses to the central retina. The macula could eventually be involved, and patients may develop cystoid macular edema and macular atrophy [7]. Our patient presented with confluent areas of chorioretinal scarring with intraretinal pigment migration and parts of the unaffected retina that appear completely normal both on fundus examination and OCT. Interestingly, the prominent area of involvement was located temporally in the right eye and nasally in the left eye, the significance of which is unknown. Initially, her central vision was preserved, but she developed progressive vision impairment as the disease advanced. No punctate white retinal opacities, fibrillar condensation of the vitreous, or anterior segment abnormalities have been found in this patient. This clinical presentation, combined with the heterozygous missense mutation in the BEST1 gene at p.(T277M), drove us to a diagnosis of ADVIRC. 

Over 200 BEST1 mutations have been documented to cause bestrophinopathies [1,6]. Of these mutations, there are currently six documented in association with ADVIRC [2,7,8]. Carter et al. (2016) reported a family with ADVIRC expressing the BEST1 missense mutation c.704T>C, p.V235A, located on exon 6 [8]. Chen et al. (2106) detailed another ADVIRC-associated mutation: a heterozygous missense mutation c.248G>A, p.G83D, located on exon 4 of BEST1 [7]. Yardley et al. (2004) reported three missense mutations in association with ADVIRC: c.256G>A, p.V86M, located on exon 4, c.715G>A, p.V239M, located on exon 7, and c.707A>G, p.Y236C, located on exon 6 [2]. Burgess et al. (2009) described another BEST1 mutation, c.707G>A, p.V235A, located on exon 6, leading to ADVIRC [9]. We believe the novel mutation reported in our patient here represents the seventh ADVIRC-associated mutation, although longer-term follow-up and more research are needed for a definite association. Notably, a very similar mutation has been described previously in association with ARB [10]. Both mutations were located on exon 7 with mutation c.830C>T, p.T277M. The only difference was the heterozygous nature of our patient’s mutation, consistent with the autosomal dominant inheritance pattern of ADVIRC, but the homozygous recessive mutation described for ARB. Although this emphasizes the close relationships of bestrophinopathies at the genetic level, the mutation presented in this case is novel due to the heterozygosity and, thus, autosomal dominant inheritance pattern, which has not been previously described for this specific mutation.

The BEST1 protein, encoded by the BEST1 gene, forms a channel in the RPE, primarily localizing to the basolateral membrane [5]. BEST1 is utilized for anion transport and regulation of intracellular volume [3,4]. Chloride is thought to be the predominant anion transported through this channel [3]. Given that the BEST1 protein channel has critical functions for maintaining RPE and general retinal health, it is obvious that noticeable pathology will develop if mutated. BEST1 mutations are thought to cause pathology through one or more of the following mechanisms: protein mistrafficking, BEST1 oligomerization defects, altered anion channel activity, and altered intracellular calcium signaling [6]. It was originally believed that BEST1 missense mutations affect pre-mRNA splicing, leading to in-frame deletion and, thus, disruption of normal protein functions [9]. However, Chen et al. (2016) failed to demonstrate that alternative splicing accounts for the underlying mechanism of the missense mutation in the first ever reported ADVIRC case in their in vitro splicing assays [7]. By using human induced pluripotent stem cell (iPSC)-derived RPE from an ADVIRC patient, Carter et al. (2016) showed that mislocalization of BEST1, as opposed to aberrant splicing, causes the pathology observed in their patient [8]. They also demonstrated that during human eye development there is a higher level of BEST1 protein expression in the peripheral compared to the central RPE, proposing that a higher level of mislocalization of BEST1 in the peripheral RPE may explain the typical phenotypes of the initial peripheral retina involvement in ADVIRC patients [8]. Besides its functions in the RPE, BEST1 plays a role in general eye development, as evidenced by additional ocular defects beyond the RPE associated with BEST1 mutations [1,6,8]. More research is warranted to elucidate the underlying mechanisms of the mutations in patients with ADVIRC.

Once the diagnosis of ADVIRC is made, it is important to convey to patients their likely prognosis and potential complications. Visual acuity impairment progresses slowly, and most patients have a good visual prognosis maintained for life [1,6,8]. The exception to this is in those with complications. Reported complications include angle closure glaucoma, retinal neovascularization, retinal fibrosis, retinal detachment, macular edema, vitreous hemorrhage, and choroid atrophy [1]. Patients in their later disease course may have iris atrophy and pseudophakodonesis [7]. Although there are no treatments available for ADVIRC currently, close follow-up is necessary to promptly manage complications as they arise. Therapies for ADVIRC and other bestrophinopathies, such as medical therapy, gene therapy, and stem cell-based RPE transplants, have been investigated, but more research is required to discover a definitive and reliable treatment plan [1,6]. 

## Conclusions

We reported a case of atypical presentation of ADVIRC with more posterior involvement, and genetic testing revealed that this case is associated with a novel missense mutation in the BEST1 gene. Furthermore, we provided a comprehensive review of the current literature regarding the documented ADVIRC mutations and suspected mechanisms of this disease. This case presentation and literature review highlight the complexity of diagnosing ADVIRC due to its non-specific presenting symptoms and variable exam findings. Regardless, when suspecting ADVIRC, it is imperative to obtain genetic testing, provide prognostic information, and follow up closely for monitoring potential complications. 
